# MHC Class II Auto-Antigen Presentation is Unconventional

**DOI:** 10.3389/fimmu.2015.00372

**Published:** 2015-07-22

**Authors:** Scheherazade Sadegh-Nasseri, AeRyon Kim

**Affiliations:** ^1^Department of Pathology, Johns Hopkins School of Medicine, Baltimore, MD, USA

**Keywords:** auto-antigens, immunodominance, HLA-DR antigens, cathepsin sensitivity, extracellular processing, paralyzed DC, cell free antigen-processing system

## Abstract

Antigen presentation is highly critical in adoptive immunity. Only by interacting with antigens presented by major histocompatibility complex class II molecules, helper T cells can be stimulated to fight infections or diseases. The degradation of a full protein into small peptide fragments bound to class II molecules is a dynamic, lengthy process consisting of many steps and chaperons. Deregulation in any step of antigen processing could lead to the development of self-reactive T cells or defective immune response to pathogens. Indeed, human leukocyte antigens class II genes are the predominant contributors to susceptibility to autoimmune diseases. Conventional antigen-processing calls for internalization of extracellular antigens followed by processing and epitope selection within antigen-processing subcellular compartments, enriched with all necessary accessory molecules, processing enzymes, and proper pH and denaturing conditions. However, recent data examining the temporal relationship between antigen uptakes, processing, and epitope selection revealed unexpected characteristics for auto-antigenic epitopes, which were not shared with antigenic epitopes from pathogens. This review provides a discussion of the relevance of these findings to the mechanisms of autoimmunity.

## Introduction

Conventional antigen presentation to CD4^+^ T cells by APCs begins by the uptake of exogenous antigens and their processing that involves transfer through a series of endosomal compartments containing suitable denaturing environment, accessory chaperones, and cathepsins (1). Antigen-processing machinery involves several accessory molecules and chaperons coevolved with proteins of major histocompatibility complex (MHC) molecules that each plays its part in epitope selection. These molecules are targeted to specialized vesicular compartments that also accommodate antigen-processing enzymes called cathepsins (2). Within the antigen-processing compartments, highly regulated pH gradient, and reducing conditions and enzymes necessary for denaturation of the antigens are available and function to optimize processing of antigen and selection of the fittest for transport to the cell membrane and presentation to T cells.

One such crucial accessory molecule/chaperon is the class II invariant chain (Ii). Newly synthesized MHC II molecule associates with Ii, which targets it to specialized endosomal compartments (MIIC) where the Ii is proteolysed by cathepsins until only a fragment known as the class II-associated invariant chain peptide (CLIP) remains bound in the MHC II peptide-binding groove. Efficient displacement of CLIP from the MHC II groove requires the accessory molecule HLA-DM in human (H2-M in mice to be called DM from now on) (3). DM functions by inducing conformational changes in pMHC II complexes, resulting in the release of the bound peptide and generation of a *peptide-receptive MHC II* (4, 5). A peptide-receptive MHC II can quickly sample a large pool of peptides derived from exogenously acquired proteins. Hence, in addition to removal of CLIP, DM helps in shaping of epitope selection. Cathepsins present in processing compartments contribute significantly by cutting and trimming of the protein antigens (processing), and gamma interferon-inducible lysosomal thiol reductase (GILT) reduces disulfide bonds in protein antigens and helps denaturation of antigen for further processing (6, 7).

Another non-classical MHC class II accessory molecule is HLA-DO, H2-O in mice, and DO for short from now on. While discovered years ago, DO has recently been shown to play an active role in peptide exchange (8–10). Of importance, DO has restricted tissue expression; it is mainly expressed in B cells and thymic medullary epithelium, where thymic deletion takes place, and certain subsets of DCs. DO cellular trafficking depends on DM. The combined efforts of all the molecules discussed above and perhaps others whose identity awaits to be discovered, leads to an impeccable selection process for very few epitopes (*immunodominant epitopes*) that occupy the MHC II groove and are transported to the APC external membrane for stimulation of CD4 T cells.

## A Cell Free System Antigen-Processing System Provides Clues to Epitope Selection

To directly address specific questions regarding steps in antigen processing and the selection of immunodominant epitopes, we have developed a reductionist antigen-processing system for MHC II molecules that incorporates defined components and accurately predicts immunodominant epitopes from protein antigens (11). The system includes soluble purified HLA-DR (DR), CatB, CatH, and CatS that process protein antigens into peptides, and DM. Mass spectrometry is used for sequencing the unique peptide peaks derived from each protein bound to the MHC II groove after cell free processing. Once the candidate epitopes are identified, their relevance to antigenicity is verified in human MHC class II bearing transgenic mice (11). Due to the defined molecular composition, this system lends itself to elucidating steps involved in antigen processing and the roles individual components play in epitope selection.

Using this minimalist system, we have discovered that the immunodominant epitopes from different antigenic sources are selected based on structural properties that allow them to form pMHC II complexes in high-relative abundance for presentation to T cells. Differential sensitivity to cathepsins and resistance to DM-mediated dissociation, equally play important roles. Unfit epitopes might be sensitive to DM-mediated peptide exchange, which results in their dissociation from the groove of MHC II, exposing them to the processing enzymes, cathepsins, and destruction. Those epitopes that fit the groove well form pMHC II complexes that are poor substrates for DM, and therefore, DM does not bind to them and displace them from the groove. Those pMHC II complexes remain intact and gain relative abundance over other epitope that when in complex with MHC II are good substrates for DM. Alternatively, a group of epitopes form complexes with MHC II that while being good substrates for DM and are displaced from the groove, are not digested away. Those epitopes are chemically resistant to cathepsin digestion. They may rebind to the MHC II groove, gain abundance, and become immunodominant epitopes. We found that the non-dominant epitopes are susceptible to both DM and cathepsins. They are often poor fits for the groove, and form DM-sensitive conformations, causing their displacement by DM. They are also sensitive to the cathepsins present in the environment, and are therefore digested away rapidly as they are dissociated from MHC II. As such, the non-dominant epitopes do not gain abundance, although they might be displayed at low-copy numbers on antigen presenting cell membranes.

## Cathepsin Sensitivity and Auto-Antigens

In general, processing of antigens cannot take place without the endocytic proteases or cathepsins. Cysteine proteases, aspartyl proteases, and serine proteases are the three major types of cathepsins studied for their roles in antigen processing (12, 13). The significance of cathepsins in antigen processing and the selection of immunodominant epitopes lies in their regulated expression levels and activity in different cell types and activation state, as well as occurrence of specific inhibitors of cathepsin activities in antigen presenting cells (14–16). Two main roles attributed to cathepsins in antigen processing are (a) to cleave off invariant chain and (b) to process antigens. Among the most extensively studied cathepsins are cathepsin (Cat)B, CatD, CatL, CatS, and asparagine endopeptidase (AEP) (17–19). CatS was reported to be involved in Ii cleavage and antigen processing (20–23) as mice deficient in CatL and CatS showed impairment of late stage invariant chain degradation in thymus and periphery, respectively (24, 25). AEP has been shown to have some role in the initial invariant chain cleavage (26), and it can either generate or destroy antigenic epitopes (27). CatB and CatD knockout mice showed some but not complete processing defect; and hence, their role in antigen processing has been considered as dispensable (28).

Our recent studies (29) have shown that inclusion of only three cathepsins, such as CatB, CatH, and CatS, was sufficient to mimic the processing conditions necessary to produce the immunodominant epitopes from several antigens. While CatB and CatH are mainly exoproteases, they also have endopeptidase activities, although the pH requirements might vary (19). We also evaluated the need for CatB in processing of two antigens in cells and observed a complete blockage of processing in the presence of a cell-permeable CatB inhibitor, CA-074ME (29). Importantly, we showed that inclusion of only CatB and CatH in our cell free antigen-processing system was sufficient for successful processing and editing of the dominant epitope of influenza HA1 epitopes, but CatS alone failed to do so on its own. It has been suggested that other groups of cathepsins, such as CatG and CatE, might also play roles in regulating antigen processing (30, 31). However, when pharmacological inhibitors of CatG or inhibitors of aspartic protease CatD, and CatE were used during the processing and presentation of type II collagen and H5N1-HA proteins in antigen presentation cell culture, we observed some reducing effects in presentation but the results were not as striking as blocking CatB (29). Therefore, while CatS, CatB, and CatH are the minimum number of processing enzymes, for a more comprehensive cell free processing system, one might benefit from further addition of CatG and CatD.

## The Relationship between Resistance to Proteases in Antigen Processing and Autoimmune Epitopes

**Figure 1:**
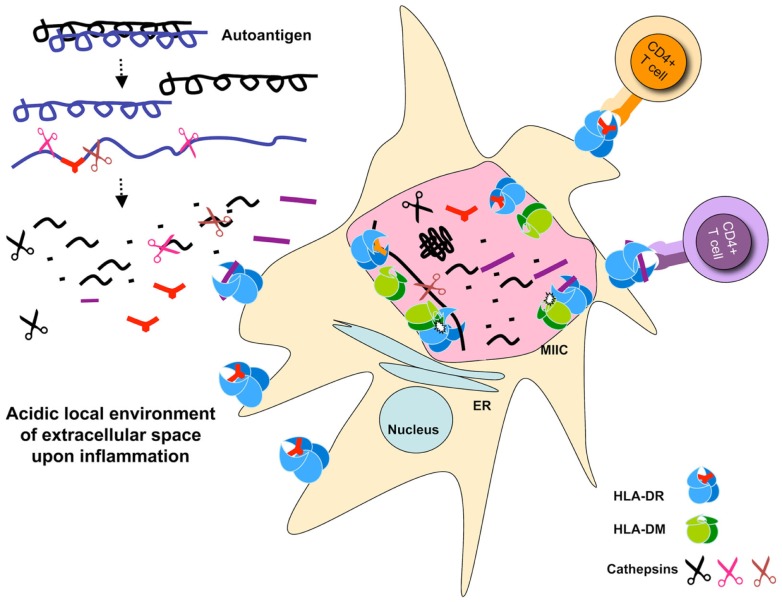
**Schematic representation of auto-antigen-processing pathways**. Auto antigen-derived epitopes are resistant to cathepsins degradation, and may or may not be sensitive to DM-mediated peptide exchange. Auto-antigens may be processed extracellularly and bind MHC II expressed on the surface of dendritic cells. Paralyzed DCs display a large number of MHC II on their surface because of exhaustive processing that occur under inflammatory conditions presumably associated with the initiation of autoimmune diseases. Processing for pathogen-derived antigens occurs in the MIIC shown as a giant pink vesicle within the DC. Cathepsins are shown as scissors, peptides, and epitopes are depicted as part of the denatured proteins, or in short stretches of sequences that carry a MHC II P1 fitting anchor or no anchor. The selected pMHC complexes are transported to the APC cell surface waiting for T cell stimulation. Small dots represent degraded peptides. Cell sizes are not depicted correctly proportionally.

As discussed earlier, all auto-antigen-derived epitopes known as targets for pathogenic autoreactive T cells we tested resisted digestion by the cathepsins in the system. While some trimming of the epitopes occurred upon long exposure to our cathepsin mix, the core MHC II binding sequences remained intact. As such, we speculate that these auto-antigens, rather than being destroyed by the processing enzymes, are likely to rebind MHC II and presented to specific T cells. By this criterion, one might hypothesize that such epitopes would not be generated in the thymus, and T cells reactive to them would be less likely to be deleted (32). In the periphery, auto-antigens are generated in extracellular matrix under inflammatory conditions where many proteases are already at work. Specifically, there are studies demonstrating that cathepsins digest various components of the extracellular matrix (33) and play an important role in the development of neurodegenerative diseases (34). Consistent with this notion under pathological conditions, such as later stages of arthritis, the pH of the local microenviroment is often found to be acidic allowing cathepsins to be active. As such, only those epitopes that survive such milieu may get a chance for presentation by APCs [see Figure 1].

There are few known examples of auto-antigens that might fall in this unusual antigen-processing pathway. First, a member of the family of matrix metalloproteinases is gelatinase B (MMP-9) that is known for its role in generating collagen II fragments (35). It is fair to say that under inflammatory conditions generated by infections or other causes, collagen fragments are generated by MMP-9 and then picked up by the APCs, which process and present them to the T cells. We have examined this epitope and found out that it is sensitive to DM-mediated dissociation. However, this epitope is resistant to the cathepsins, and as such, collagen II epitope can rebind the MHC II groove and is presented to T cells. A second example is the dominant epitope of thyroglobulin, which has been reported to induce autoimmune thyroid diseases (AITD). It is documented that this epitope is generated from thyroglobulin in the thyroid tissue by multiple cathepsins at neutral pH and then is taken up by the APCs (36, 37). We showed that the DR3 binding core of this epitope is resistant to further cleavage by the cathepsins. A third example is a experimental autoimmune uveitis (EAU) inducing peptide, which when exposed to CatS, CatB, and CatH mixture it does not lose its effectiveness in inducing EAU in DR3 transgenic mice (38). Both EAU and thyroglobulin dominant epitopes were resistant to DM-mediated dissociation, suggesting that auto-antigens may or may not be DM-sensitive. A forth example is a DR2 restricted MBP(84–102) peptide, which follows the same trend as the other auto-antigens tested. A report has shown that MBP can be cleaved by CatS, although the major cutting sites fall outside the core binding site of the immunodominant MBP(84–102) epitope (39). A fifth example is in celiac disease, an autoimmune disease of the small intestine caused by exposure to dietary gluten prevalent among individuals expressing HLA-DQ2 or HLA-DQ8. Ingestion of gluten induces an inflammatory response leading to the destruction of the villous structure of the intestine. The toxic components of glutens are a family of closely related proline and glutamine rich proteins called gliadin. Shan et al. identified the dominant epitope of gliadin and showed its extreme resistance to digestion by gastric and pancreatic enzymes (40, 41).

In all, it seems that unlike pathogen-derived epitopes, auto-antigens rely on resistance to enzymatic digestion as key determinant of immunodominance, and while DM resistance can be an added as an advantage, it is not an absolute necessity. As such, it is likely that during inflammation auto-antigens are processed extracellularly, and binds MHC class II molecules displayed on the surface of paralyzed DC known for displaying large numbers of pMHC II on their surface (42).

## Prevalence of Multiple Registers for Autoimmune Epitopes and Resistance to Cathepsins

Several well-characterized auto-antigens have been reported to have multiple registers, i.e., peptide can fit the MHC II groove using two or more sets of anchoring amino acids. An excellent example is MBP(89–101) peptide that has two registers for binding to DR2a and DR2b (43, 44). When MBP(89–101) peptide was tested for cathepsin sensitivity in our cell free antigen-processing system, although some cutting occurred, the resulting new peptides could fit in the two registers defined and demonstrated by crystal structures of DR2a or DR2b. Interestingly, in agreement with our model for immunodominance, MBP(89–101) epitope is sensitive to DM-mediated dissociation (45), and as per our own experimental data, it is insensitive to cathepsins (29). Accordingly, it is likely that MBP dominant epitope that is the target of MBP-specific autoreactive T cells binds some empty DR2 molecules expressed on APCs.

Similar to MBP(89–101) epitope, digestion of insulin B7–23 epitope by the cathepsins mixture did not result in its destruction (29). The only other fragment detected post digestion of insulin B7–23 was a previously described I–A^g7^ binding epitope (32, 46–48). Of interest is that insulin B7–23 peptide has also been reported to have at least two registers for binding to I–A^g7^. In one register, insulin B7–23/I–A^g7^ complex can only be recognized by Type-A T cells, which can recognize epitopes processed within the conventional antigen-processing pathway, i.e., peptide binding should occur in the presence of DM. The type-A T cell I–A^g7^ binding register is resistant to DM-mediated peptide exchange. The other peptide register forms complexes with MHC II that readily dissociate by DM, and hence, they are DM-sensitive. In the absence of DM resistance, such complexes stimulate Type-B T cells and trigger their autoreactivity causing type I diabetes, as suggested by Unanue and colleagues (49, 50). While we have discussed only two well-studied peptides known for induction of autoreactivity and for having more than a single registers, other peptides that cause autoimmune diseases are likely to fit this criterion.

## Conclusion

We have discussed molecular mechanisms that foster the induction and development of autoimmune diseases using a minimalist cell free antigen-processing system. Abundant quantities of auto-antigens released due to tissue insults during inflammation, as well as the presence of a variety of proteolytic enzymes lead to generation of epitope fragments extracellularly. Insensitivity to further degradation by the processing enzymes appears as the key characteristic of auto-antigens. Inflammatory conditions and continuous processing of self-antigens by tissues resident dendritic cells may lead to their paralysis. Paralyzed DCs display large numbers of pMHC II on their surface some of which might exchange their peptides with the extracellularly processed epitopes from auto-antigens and emerge as targets for autoreactive T cells.

## Conflict of Interest Statement

The authors declare that the research was conducted in the absence of any commercial or financial relationships that could be construed as a potential conflict of interest.
